# Genome Sequence of a Novel Iflavirus from mRNA Sequencing of the Butterfly *Heliconius erato*

**DOI:** 10.1128/genomeA.00398-14

**Published:** 2014-05-15

**Authors:** Gilbert Smith, Aide Macias-Muñoz, Adriana D. Briscoe

**Affiliations:** Department of Ecology and Evolutionary Biology, University of California, Irvine, Irvine, California, USA

## Abstract

Here, we report the genome sequence of a novel iflavirus strain recovered from the neotropical butterfly *Heliconius erato*. The coding DNA sequence (CDS) of the iflavirus genome was 8,895 nucleotides in length, encoding a polyprotein that was 2,965 amino acids long.

## GENOME ANNOUNCEMENT

*Heliconius* butterflies are a diverse group of around 40 neotropical species that are widely studied for their wing color pattern diversity and Müllerian mimicry, and display a striking pattern of both divergent and convergent evolution in wing phenotypes (1, 2). *Heliconius erato* is native to Central and South America where a huge diversity of wing phenotypes are expressed across races from different geographic locations (3). Iflaviruses are single-stranded RNA viruses that infect insect hosts, often leading to developmental problems and the death of the host (4). However, little is known about the viral (and bacterial) assemblages infecting *Heliconius* butterflies.

We sequenced the mRNA of one male and one female of *H. erato*, obtained from a Costa Rican butterfly farm, Suministros Entomológicos Costarricenses, S.A. RNA was extracted from two individuals for each of four tissue types (the antenna, mouth-parts, head, and legs) using TRIzol (Life Technologies). RNA sequencing libraries were prepared using the mRNA-Seq sample preparation kit (Illumina) and sequenced using an Illumina HiSeq 2000 sequencer by the UCI Genomics High-Throughput Facility. In total, eight libraries were sequenced, producing an average library size of approximately 24 million 100-bp paired-end reads, after quality control filters were applied. Paired-end libraries were then *de novo* assembled into putative mRNA transcripts using Trinity (5). The Trinity-assembled nucleotide (nt) contigs were translated into amino acid (aa) sequences, and a BLASTp comparison to the NCBI NR protein database (6) revealed the presence of a viral polyprotein.

The best BLASTp hit for the discovered viral polyprotein matched a strain of iflavirus previously described from the Chinese oak silkmoth, *Antheraea pernyi*. The *H. erato* iflavirus polyprotein amino acid sequence demonstrated a 63% pairwise similarity to the Chinese oak moth iflavirus polyprotein. The coding DNA sequence (CDS) of the *H. erato* iflavirus was 8,895 nucleotides in length, encoding a 2,965-aa polyprotein. The entire iflavirus genome was 9,910 nucleotides long, which included a 906-nt 5′ untranslated region (UTR) and 110-nt 3′ UTR, and had a G+C content of 35.3%.

Iflavirus genomes typically encode a single polyprotein that is comprised of conserved domains for structural proteins in the N-terminal region, and non-structural proteins, RNA helicase, protease, and RNA-dependent RNA polymerase (RdRp) proteins in the C-terminal region (4). The 2,965-aa polyprotein of the *H. erato* iflavirus strain contained an arrangement of conserved domains common to that of previously described insect iflavirus genomes (7). This included two rhv-like picornavirus capsid protein domain-like motifs (Pfam entry, cd00205), an RdRp motif (Pfam entry, cd01699), RNA helicase (Pfam entry, PF00910), a cricket paralysis virus (CRPV) capsid protein-like motif (Pfam entry, PF08762), and a 3-C cysteine protease motif (Pfam entry, PF00548).

We present the complete genome of a novel strain of iflavirus isolated from *H. erato*. This *H. erato* iflavirus genomic sequence encodes a full and complete polyprotein when compared to other iflavirus genomes. The increasing availability of mRNA sequencing data from a wide range of insect species promises to aid in the understanding of the ecology and evolution of insect viral infection.

### Nucleotide sequence accession number.

The genome sequence was deposited at DDBJ/EMBL/GenBank under accession no. KJ679438.
